# Effect of Supervised and Unsupervised Exercise Training in Outdoor Gym on the Lifestyle of Elderly People

**DOI:** 10.3390/ijerph20217022

**Published:** 2023-11-06

**Authors:** Welmo A. Barbosa, Carine Danielle F. C. Leite, Carlos H. O. Reis, Alexandre F. Machado, Valentina Bullo, Stefano Gobbo, Marco Bergamin, Ana Paula Lima-Leopoldo, Rodrigo L. Vancini, Julien S. Baker, Roberta L. Rica, Danilo S. Bocalini

**Affiliations:** 1 Experimental Physiology and Biochemistry Laboratory, Physical Education and Sport Center, Federal University of Espírito Santo, Campus Goiabeiras, Avenida Fernando Ferrari, 514, Goiabeiras, Vitória 29075-910, ES, Brazilbocaliniht@hotmail.com (D.S.B.); 2 MoveAgeLab, Physical Education and Sport Center, Federal University of Espírito Santo, Vitória 29075-010, ES, Brazil; 3 Department of Medicine, University of Padova, 35122 Padova, Italy; 4 Center for Health and Exercise Science Research, Department of Sport, Physical Education and Health, Hong Kong Baptist University, Kowloon Tong 999077, Hong Kong; 5 Department of Physical Education, Estacio de Sá University, Vitoria 22640-102, ES, Brazil

**Keywords:** aging, physical exercises, lifestyle, outdoor equipment

## Abstract

The aim of this study was to investigate the effectiveness of supervised and unsupervised physical training programs using outdoor gym equipment on the lifestyles of elderly people. Methods: physically independent elderly people were randomly distributed into three groups: supervised training (n: 20; ST), unsupervised training (n: 20; UT) and control (n: 20; C). The ST and UT groups completed a 12-week program, with exercises performed three times a week. The ST group underwent weekly 30 min sessions consisting of a 5 min warm-up (walking at 60% of HR_max_), followed by 20 sets of 30, “monitored by a metronome with 30” of passive recovery between sets and a five-minute cool-down. The following equipment was used: elliptical, rowing, surfing and leg press. The UT group was instructed to freely attend the gym and train spontaneously using the same equipment used by ST. Lifestyle changes were evaluated using a questionnaire containing specific domains. Results: no significant differences were identified in the domains for family, physical activity, nutrition, smoking, sleep, behavior, introspection, work and overall score; however, the values corresponding to the alcohol domain for the ST and UT groups were lower (*p* < 0.05) than the C group, remaining even lower after the 12 weeks of intervention. Time effect (*p* < 0.05) was found only in the ST group for the physical domains, sleep, behavior and overall score. Conclusion: elderly people submitted to supervised and unsupervised physical exercise programs using outdoor gym equipment present positive changes in lifestyle parameters compared to physical inactive elderly people.

## 1. Introduction

Aging has been a globally investigated phenomenon over the past decades. The increase in life expectancy brings about demographic, epidemiological, and technological impacts for the elderly population in both developed and developing countries [1]. Hence, the need for specialized care for this population, aiming at improving quality of life and promoting lifestyle enhancement strategies, is growing [1]. 

Additionally, it is known that lifestyle directly impacts the longevity and quality of life in the elderly population. Therefore, inappropriate behaviors such as sedentary lifestyle, physical inactivity, and inadequate nutrition are discouraged, primarily due to their association with detrimental effects on health indicators [1]. There are still barriers to the elderly’s access to health promotion methods. These barriers can be linked to various environmental, social, financial, and personal factors, which can adversely affect the physical, physiological, and mental health of the participants [2]. Evidence has suggested that adopting healthy habits as one ages, including engaging in regular physical activity, can contribute to improvements in physical, functional, and cognitive fitness conditions, as well as playing a role in preventing mood disorders such as depression and anxiety in elderly individuals [3,4].

Information regarding inappropriate lifestyle behaviors among the elderly population suggests that they have detrimental effects on health. Therefore, the implementation of strategies and programs aimed at health-care-related actions, as well as initiatives promoting lifestyle changes for the elderly, are essential. From this perspective, in Brazil, there are numerous physical activity programs for the elderly [5]. Programs using popular public gyms for the elderly (APPIs) offer easily accessible gym equipment installed in public places [6,7], which allows for the practice of physical exercise. It is important to highlight here that the practice can occur spontaneously or be guided by a physical education professional.

Several authors emphasize the value of popular gyms in promoting the health of the Brazilian population, particularly due to their free access, which potentially facilitates entry for individuals facing challenges in adhering to physical exercise [7,8]. Thus, the enhancement of functional capacity resulting from movement provides the elderly with the independence to make decisions and engage in their daily lives, generating significant impacts on quality of life [9]. 

Popular gyms consist of gym equipment installed in public places [6], with the aim of promoting physical activity, social inclusion, improvements in self-esteem and overall health among the elderly [10]. Researchers have aimed to investigate the profile of visitors to these spaces [6,11,12]. The literature reports only two studies that examine the effects of the long-term use of this equipment through intervention [13,14]; however, there was no measurement of lifestyle changes. 

Considering that popular gyms may promote increments in health indicators of populations and easily to facilitate adherence to active behavior, to the best of our knowledge, there is little information about the correlation between using public gym equipment and lifestyle outcomes in the elderly. Thus, given the lack and inconsistency of studies about the effects of long-term exercise using senior gym equipment on lifestyle changes, this study aimed to verify the effectiveness of a supervised physical training program using equipment from APPIs on the lifestyle of the elderly. Additionally, to increase the internal consistency of the study, a group of older adults who trained without supervision were also analyzed with the hypothesis that supervised practice would promote better results for the evaluated parameters.

## 2. Materials and Methods

After ethical approval was received from the Research Committee of the Federal University of Espírito Santo (number n° 4.088.540-2020), physically independent elderly individuals (>60 years) were invited to participate voluntarily in this study. The invitation was made in community centers and fitness centers, and by posters fixed in the squares and parks near senior gyms. The study sample was considered an intentional non-probabilistic sample, selected for convenience with the experimental design.

The following exclusion criteria were applied: recent hospitalization, symptomatic cardiorespiratory disease, uncontrolled hypertension or metabolic syndrome, severe renal or hepatic disease, cognitive impairment, progressive and debilitating conditions, severe obesity with inability for physical activity, recent bone fractures or any other medical contraindications for exercise practice. 

Initially, 80 elderly individuals volunteered to participate in the project; however, after applying the exclusion criteria, 60 elderly individuals were deemed eligible for participation. After randomization of volunteers and distribution of the signed informed consent form, in accordance with the Declaration of Helsinki, the elderly participants were distributed into three groups:

1. Supervised Trained (ST, n: 20; 66 ± 5 years old): Elderly participants subjected to training with controlled cadence and supervised by a Physical Education professional throughout the training program.
2. Unsupervised Trained (UT, n: 20; 68 ± 7 years old): Elderly participants who exercised freely without the supervision of a Physical Education professional throughout the program.
3. Control (C, n: 20; 68 ± 5 years old): Elderly participants who did not engage in exercise but received support to maintain their daily routines throughout the entire investigation period.

The sample size required was estimated using G* Power 3.1 software (version 3.1.9.4). A priori power analysis assuming an estimating error of α + 0.05, and power = 80% to an actual power was 0.80. The randomization process occurred in blocks of six subjects, with each block resulting in the distribution of two subjects per group, ensuring balanced recruitment in the study and promoting similarity of measures at the initial stage among the groups. This strategy reduces the risk of bias, which is considered a quality criterion in experimental designs aiming to make group comparisons. 

The elderly participants underwent training sessions in a public square located in the city of Vitória, Espírito Santo, Brazil. All individuals conducted activities at the same time of day as a means of adapting to the climate and controlling collected data for contamination due to diurnal variation. Additionally, they underwent familiarization sessions with the equipment to correct movements and reduce the risk of accidents or injuries. Only the elderly individuals who achieved a minimum attendance rate of 85% in the training sessions were included in the analysis.

### 2.1. Intervention

The ST and UT groups underwent a 12-week exercise program, conducted 3 times a week for 30 min. Subjects in the supervised training group had weekly 30 min sessions, comprising 5 min of warm-up (walking at 60% of the maximum heart rate monitored by a Polar (H10) heart rate monitor), followed by 20 sets of 30 s each using a moderate cadence (1 movement every 2 s), controlled by a metronome, with 30 s of passive recovery between sets and 5 min of cool-down, on non-consecutive days. The UT group was instructed to freely visit the space; however, they exercised spontaneously according to the APPIs program recommendation, using only the equipment proposed by the study. The equipment utilized in this study included an elliptical rowing machine, surfboard, and leg press, as per a previous study [15]. Additionally, the control group was advised to maintain their daily routines throughout the entire investigation period.

### 2.2. Evaluated Parameters

#### Lifestyle Questionnaire

The assessment of lifestyle was conducted using the validated “Fantastic Life Questionnaire” for Brazilian populations [16], administered and answered by the participants. The “Fantastic Life Questionnaire” is a self-administered instrument that considers behaviors exhibited by individuals in the last month, and the results are obtained by associating lifestyle with health itself. The tool comprises 25 questions, divided into nine domains: family and friends, physical activity, nutrition, smoking, alcohol consumption, illicit drug use, sleep, seatbelt usage, stress, safe sex, type of behavior, introspection, and work. 

At the end of the collection, a score was obtained, which categorized individuals into five categories: “Excellent” (85 to 100 points), “Very Good” (70 to 84 points), “Good” (55 to 69 points), “Fair” (35 to 54 points), and “Needs Improvement” (0 to 34 points). It was considered desirable that participants reached at least the “Good” classification, given that the lower the individual’s score, the greater the need for lifestyle changes.

In this way, it is understood that individuals who achieved the “Excellent” classification were established as those who have a lifestyle with excellent health benefits; “Very Good” indicates a lifestyle that promoted very good health benefits; “Good” signifies that their lifestyle yielded many health benefits; “Fair” implies that in some way the lifestyle provided some health benefit, although health risks are also present; “Needs Improvement” represents the lowest score, indicating that significant changes are necessary, as this lifestyle carries many risk factors for the individual’s health. 

### 2.3. Statistical Analysis

The Shapiro–Wilks test was used to assess normality. To evaluate the effect of time and intervention, a repeated measure analysis of variance (ANOVA) was employed with two factors: time (pre- and post-intervention) vs. group (trained and untrained), with post hoc Bonferroni analysis conducted where appropriate. The Chi-square test was used to assess categorical parameters. The effect size was calculated using Cohen’s d. The following interpretation was utilized: <0.01 = small effect; 0.06 = moderate effect; and ≥0.14 = large effect. The data are presented as mean ± standard deviation, and all statistical analyses were conducted using GraphPad Prism software (version 4.0, San Diego, CA, USA), adopting a significance level of 5%.

## 3. Results

During the intervention period, five older adults from the control group did not provide results and three indicated health problems that prevented the second evaluation from being conducted. Regarding the UT group, fourteen individuals completed the intervention period, with two individuals reporting a lack of motivation and dropping out of the project, and a further four individuals did not meet the minimum attendance requirement. Regarding the ST group, no changes in the number of subjects were identified during the intervention period.

No significant differences were identified in the domains of family, physical activity, nutrition, smoking, sleep, behavior, introspection, work and overall score. However, the values corresponding to the alcohol consumption domain in the ST and UT groups were significantly lower than those in the C group, and this result was maintained after the 12-week intervention, as described in Table 1. 

There was only a time effect in the ST group in the domains of physical activity (F = 0.5394; *p* = 0.0066), sleep (F = 1.022; *p* = 0.0216), behavior (F = 0.395; *p* = 0.0095) and overall score (F = 4.270; *p* < 0.0001). However, only the sleep parameters (F = 1.022; *p* = 0.03683) and overall score (F = 4.270; *p* = 0.0204) exhibited a significant interaction effect after the 12-week intervention.

**Figure 1:**
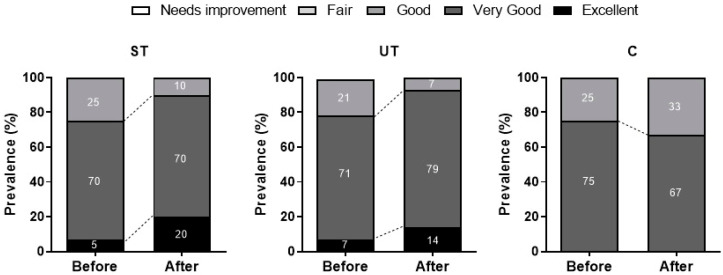
Lifestyle classification of the supervised training group (ST), unsupervised training group (UT) and control group (C) after 12 weeks of intervention.

Considering the classification of lifestyle before and after the 12-week intervention (Figure 1), no significant changes were found among the ST group (χ^2^: 3.086, *p* = 0.2138), UT group (χ^2^: 1.381, *p* = 0.5013), and C group (χ^2^: 0.2017, *p* = 0.9041).

## 4. Discussion

The current study aimed to investigate the effects of a 12-week training program, both supervised and unsupervised, using equipment from popular gyms for elderly individuals to measure lifestyle parameters. It is well understood that lifestyle directly affects the longevity and quality of life of the elderly population [17]. Lifestyle is characterized as a collection of habits, choices, and practices experienced by individuals throughout their lives [18]. 

One noteworthy aspect of this study is the family dynamic, as the elderly person’s interactions within the family environment extend their social support, directly impacting mental health and contributing to the quality of life of the elderly [19,20]. However, no significant differences were observed among the assessed groups in terms of the family domain. It is known that family encouragement of exercise is essential for facilitating changes in the elderly population’s lifestyle. Thus, Pinheiro and Coelho Filho [6], when analyzing the lifestyle of 374 elderly individuals attending a senior’s gym, demonstrated that nearly two thirds of their respondents received encouragement to physical exercise. Another study reported that 58.6% of attendees received some level of familial social support [21]. Although these findings appear favorable, further studies are still necessary to comprehensively investigate this matter.

As anticipated in our study, the groups engaging in physical activity demonstrated improved scores after the 12-week program. Recognizing physical activity as a crucial element in lifestyle change and mortality, consequently, it is understood that regular exercise practice has an impact on the survival, well-being and quality of life of the elderly, regardless of gender, age, health parameters and lifestyle [17,18]. 

Simultaneously, nutrition contributes to significant changes in the lifestyle of the elderly, promoting an enhanced perception of quality of life [17,18]. In the present study, we did not identify significant differences among the groups; however, it is worth noting that there was no dietary control proposed during the intervention. The absence of dietary recommendations for the elderly population can compromise certain outcomes. Despite adequate physical exercise, adaptations to training may not be satisfactory due to an inadequate diet that fails to meet individual needs [22]. Therefore, this domain necessitates individualized monitoring under the supervision of a specialized professional, emphasizing the importance of dietary control and/or the recommendation of dietary supplements [23]. 

Among several habits that can help achieve a better lifestyle, studies indicate that the absence of tobacco consumption is associated with a better perception of quality of life among the elderly [19,24]. Smoking is a chronic habit that, when developed, becomes associated with nicotine dependence [25]. The study by Marques et al. [20], assessed the lifestyle of 383 elderly individuals from the municipality of Juiz de Fora, Minas Gerais, using a sociodemographic questionnaire to analyze lifestyle profiles. The authors identified that non-smokers had a better lifestyle compared to smokers, as expected. Although the risks associated with tobacco use are widely known, the number of smokers remains significant, and consequently, they are more prone to health issues [26,27].

Regarding alcohol consumption, the present study identified that although the results did not show a statistically significant effect, the trained groups demonstrated lower alcohol consumption compared to the control group. Alcohol consumption is a contributing factor to numerous diseases and is known to be associated with the development of chronic conditions [28,29]. A study demonstrating an association between physical activity and alcohol consumption indicated that men were more likely to consume alcohol [30]. This trend of higher alcohol consumption among men is prevalent in the elderly population, as demonstrated in a previous investigation [31]. Considering these findings, a higher prevalence of a healthy lifestyle is observed among women [32]. These findings align with studies that indicate men are more susceptible to diseases and have a shorter life expectancy compared to women [33].

Regarding sleep in the elderly, the present study demonstrated that ST and UT groups differed from the C group. It is known that sleep is a physiological and heterogeneous event responsible for the restoration of the body, and physical activity is a therapeutic and preventive approach capable of regulating the sleep cycle without side effects, contributing to the maintenance of physical and emotional balance [34]. Sleep quality is indeed related to overall wellness in life. It is important to understand that lifestyle can also influence sleep quality [18]. When considering factors such as physical exercise and nutrition in association with lifestyle, these components can have impacts on sleep quality and consequently influence the overall quality of life for the elderly [35,36]. Freire et al. [34] investigated the sleep quality of elderly individuals who underwent four weeks of hydro-gymnastics practice, using the Pittsburgh Sleep Quality Index questionnaire administered before and after the four-week period. The study demonstrated a 20% increase in the parameter ‘good sleep quality’ and a 15% reduction in the ‘poor sleep’ parameter. Other researchers, also using the Pittsburgh Sleep Quality Index questionnaire, subjected 118 elderly individuals to a twelve-week physical activity program, demonstrating the maintenance of sleep quality in active elderly individuals, while the control group showed a worse score, showing the presence of sleep disorders [37].

Thus, it appears to us that physical exercise has the potential to maintain or improve the sleep quality of active elderly individuals. Sleep quality is crucial throughout life; therefore, observing sleep patterns is essential. Disruption in regular sleep patterns is present in approximately 8–18% of the general population and around 50–70% of the elderly population [38]. This is a common characteristic in the elderly population, considering that the aging process brings about changes in the regular sleep pattern, leading to a decrease in its quality [39]. 

Regarding the behavior of the elderly, only the ST group showed modifications after 12 weeks of training. The “Fantastic Lifestyle Questionnaire” considers the following behaviors in by asking participants how often they are “feeling rushed and feeling angry and hostile”, with the options of choice being “Almost always”, “Fairly often”, “Sometimes”, “Rarely” and “Almost never”. Lifestyle-related behaviors are important for improving the quality of life of this population [18]. It is known that, with aging, it is necessary for certain behaviors to be adopted to counteract the deleterious effects among the elderly, and changes such as a balanced diet and physical activity can mitigate this process [40]. It is important to highlight that lifestyle changes are essential for maintaining and improving the health of elderly populations, and the harmful effects of the aging process can be delayed with these types of behaviors, as already evidenced [41,42].

Over the years, everyone tends to become somewhat more introspective; however, in our findings, no significant changes were evident among the participating groups. Studies indicate that this factor is part of the process of change in the lives of the elderly [43]. Considering this factor, Cerqueira and Ulian [22], using the Fantastic Lifestyle Questionnaire to assess the lifestyle of elderly individuals engaged in weightlifting, observed that the majority exhibited positive and optimistic thoughts. Studies indicate that optimistic thoughts, as well as physical activity, are positive factors, as mental health is of the utmost importance in individuals’ quality of life, thus preventing associations with suicide, depression, and anxiety [40,43]. 

In the present study, the work domain did not show significant differences among the evaluated groups. According to Marques et al. [44], work does not interfere with individuals’ lifestyle. As shown by Antunes and More [45], retirement for some elderly individuals can evoke mixed feelings, as many of them fear idleness and losing a sense of purpose in life. Supporting these findings, Cerqueira and Ulian [22] demonstrated that the elderly participants in their study reported satisfaction with work, where 60% expressed contentment, given that a work routine holds significant importance in the social context, including the factors of livelihood and well-being maintenance. Work is a factor that encompasses more than just income; it involves labor practices, reinforces identity, self-worth, and personal development. Additionally, it serves as a health-promoting activity, enabling greater social integration, independence and autonomy [46]. In this context, research highlights the significance of labor-related work for the health of the elderly, emphasizing the association between work and the maintenance of elderly individuals’ health. Retirement is linked to declines in physical, psychological, and cognitive abilities, as well as impairments in autonomy, independence, social support and interpersonal relationships [47,48]. Therefore, work serves as a means for the elderly to stay active, contributing to mental health and social relationships. It acts as a positive tool in their lifestyle, consequently impacting the overall quality of life for the elderly [49].

Finally, Figure 1 illustrates the analysis of the questionnaire among the groups (ST, UT, and C) regarding the % prevalence before and after the 12 weeks of training. Positive outcomes were observed among the supervised and unsupervised training groups, indicating that, overall, the subjects displayed an adequate lifestyle with scores ranging from “Good” to “Excellent”, in contrast to the non-training group. Our findings are consistent with other studies involving physical activity practitioners who demonstrated an appropriate lifestyle according to the classification of the administered questionnaire [44,49]. Thus, our results emphasize the importance of engaging in physical exercise, whether supervised or unsupervised, for maintaining a positive lifestyle among the elderly.

Some limitations should be considered, such as the low level of physical activity among older adults, the intervention time, the monitoring/control of calorie intake and functional measures that do not allow for generalizations. Although the evaluation of lifestyle has been investigated by questionnaire, an objective evaluation, mainly of physical activity, is necessary. Additionally, it is important to consider the reliability or internal consistency of the instrument, which was not evaluated. Finally, senior gyms present a considerable variety of equipment and, therefore, our results should not be applied in other exercise/equipment or session designs, which does not allow for generalizations of our findings.

## 5. Conclusions

In conclusion, based on our findings, elderly individuals subjected to physical exercise programs exhibit a better lifestyle compared to inactive elderly individuals. However, certain limitations should be acknowledged in this study, as the results were obtained by accessing elderly attendees of APPIs, which may not represent other forms of physical exercise and/or different environments. In general, the lifestyle of elderly individuals engaged in physical activity, both supervised and unsupervised, at APPIs was deemed satisfactory. These findings underscore the significance of engaging in physical activity, as it yields numerous beneficial impacts on the health of this population. Furthermore, the use of the questionnaire proved to be a valuable tool for diagnosing the lifestyle of the elderly, enabling the Physical Education professional to gain deeper insights into their experimental subjects and to positively contribute to interventions in the health and quality of life of elderly individuals.

## Figures and Tables

**Table 1:** Effect of 12 weeks of training in popular gyms on elderly lifestyle parameters.

Domain	Before	After	MD	95% IC	ES	ANOVA		
						Time	TimexGroup	
						*p*	F	*p*
Family								
ST	5.60 ± 2.39	6.15 ± 1.95	−0.55	−1.118–0.017	0.23	=0.0606	0.3430	=0.0711
UT	5.85 ± 2.44	6.35 ± 1.86	−0.50	−1.179–0.178	0.20	=0.2203		
C	5.83 ± 2.48	6.08 ± 2.39	−0.25	−0.983–0.483	0.10	>0.9999		
Physical activity								
ST	4.90 ± 2.07	6.10 ± 1.41	−1.20	−2.118–−0.282	0.57	=0.0066	0.5394	=0.5870
UT	4.92 ± 1.97	5.64 ± 1.86	−0.71	−1.811–0.382	0.36	=0.3362		
C	4.66 ± 2.10	5.33 ± 1.61	−0.66	−1.852–0.518	0.31	=0.5045		
Nutrition								
ST	8.80 ± 1.85	9.25 ± 2.14	−0.45	−0.992–0.092	0.24	=0.1341	1.133	=0.3315
UT	9.21 ± 1.62	9.50 ± 1.95	−0.28	−0.933–0.362	0.17	=0.8343		
C	9.08 ± 2.35	9.00 ± 2.25	0.08	−0.616–0.783	0.03	>0.9999		
Smoking								
ST	13.95 ± 1.73	14.05 ± 1.79	−0.10	−0.391–0.191	0.05	>0.9999	2.219	=0.1211
UT	14.21 ± 1.12	14.35 ± 1.21	−0.14	−0.491–0.205	0.12	=0.9376		
C	14.00 ± 1.70	13.75 ± 1.81	0.25	−0.126–0.626	−0.14	=0.3152		
Alcohol								
ST	11.75 ± 1.11	11.80 ± 0.89	−0.05	−0.260–0.160	1.11	>0.9999	2.981	=0.0613
UT	11.64 ± 1.33	11.71 ± 1.06	−0.07	−0.322–0.179	0.05	>0.9999		
C	14.00 ± 1.70	13.75 ± 1.81	0.25	−0.0215–0.521	−0.15	=0.0802		
Sleep								
ST	13.40 ± 0.13	14.35 ± 2.08	−0.95	−1.789–−0.111	0.44	=0.0216	1.022	=0.0368
UT	13.71 ± 0.05	14.42 ± 1.86	−0.71	−1.717–0.288	0.34	=0.2489		
C	13.16 ± 2.69	13.33 ± 2.87	−0.16	−1.249–0.916	0.06	>0.9999		
Behavior								
ST	4.00 ± 2.20	4.75 ± 1.94	−0.75	−1.348–−0.152	0.34	=0.0095	0.395	=0.6758
UT	3.35 ± 2.30	4.07 ± 2.16	−0.71	−1.429–0.000	0.31	=0.0501		
C	3.16 ± 1.99	3.58 ± 1.56	−0.41	−1.188–0.355	0.21	=0.5571		
Introspection								
ST	7.60 ± 2.47	7.90 ± 2.65	−0.30	−0.780–0.180	0.12	=0.3812	0.2661	=0.7676
UT	7.92 ± 2.46	8.21 ± 2.66	−0.28	−0.859–0.288	0.11	=0.6654		
C	7.25 ± 2.09	7.33 ± 1.77	−0.08	−0.703–0.536	0.03	>0.9999		
Work								
ST	3.60 ± 0.50	3.70 ± 0.47	−0.10	−0.388–0.188	0.20	>0.9999	0.2575	=0.7742
UT	3.57 ± 0.51	3.71 ± 0.46	−0.14	−0.487–0.201	0.27	=0.9222		
C	3.58 ± 1.16	3.58 ± 0.79	0.00	−0.372–0.372	0.00	>0.9999		
Overall score								
ST	73.60 ± 7.07	78.05 ± 6.69	−4.45	−6.222–−2.678	0.62	<0.0001	4.270	=0.0204
UT	74.42 ± 6.59	78.00 ± 5.69	−3.57	−5.690–−1.453	0.54	=0.0004		
C	72.91 ± 6.35	74.00 ± 5.08	−1.08	−3.371–1.205	0.17	=0.7341		

Values are expressed as mean ± standard deviation for the supervised training group (ST), unsupervised training group (UT), and control group (C) after training in popular gyms for elderly lifestyle parameters. 95% CI: 95% confidence interval. ES: effect size.

## Data Availability

Data presented in the current paper are available upon request.
